# Factors influencing interprofessional collaboration in general and during multidisciplinary team meetings in long-term care and geriatric rehabilitation: a qualitative study

**DOI:** 10.1186/s12909-024-05291-8

**Published:** 2024-03-15

**Authors:** Arno J. Doornebosch, Wilco P. Achterberg, Hanneke J. A. Smaling

**Affiliations:** 1 Department of Public Health and Primary Care, Leiden University Medical Center, Leiden, The Netherlands; 2 University Network for the Care sector Zuid-Holland, Leiden University Medical Center, Leiden, the Netherlands

**Keywords:** Interprofessional collaboration, Long-term care, Geriatric rehabilitation, Multidisciplinary team meetings, Facilitators, Barriers, Patient outcome measures

## Abstract

**Background:**

Interprofessional collaboration is essential to maintain high-quality care in long-term care and geriatric rehabilitation. However, little is known regarding perceived factors influencing interprofessional collaboration by people involved in care. This concerns both long-term care and geriatric rehabilitation. Moreover, knowledge of using patient outcome measures to enhance interprofessional collaboration during multidisciplinary team meetings is insufficient. This study examined the perceived facilitators of and barriers to interprofessional collaboration in general and during multidisciplinary team meetings, specifically according to healthcare professionals, patients, and informal caregivers. Differences between long-term care and geriatric rehabilitation were also investigated. Finally, it was examined which patient outcome measures were used in multidisciplinary team meetings.

**Methods:**

A constructivist qualitative study using 10 focus groups and 18 semi-structured interviews with 14 patients, 13 informal caregivers,10 managers, and 22 healthcare professionals from eight Dutch long-term care and geriatric rehabilitation facilities. A combined inductive and deductive approach to a thematic analysis was performed.

**Results:**

The perceived influencing factors of interprofessional collaboration were classified into two general themes: (1) ‘Involvement of patient, informal caregiver, and healthcare professional’, categorised into: ‘participation of patients and informal caregivers’, ‘behaviour and attitude of team members’, ‘expectations of team members towards each other’, and ‘exchange of information, knowledge, and reciprocity in communication’; and (2) ‘A systematic approach to providing care for older people’, consisting of: ‘coordination of team procedures’, and ‘coordination of organisational procedures’. Also, one theme for multidisciplinary team meetings was identified: ‘Organised participation of patient, informal caregiver, and healthcare professional in multidisciplinary team meeting, categorised into: ‘team procedures’, ‘working systematically’, and ‘participation in multidisciplinary team meetings. Standardised patient outcome measures were scarcely used in multidisciplinary team meetings.

**Conclusion:**

People involved in long-term care and geriatric rehabilitation indicated that, apart from working systematically, being involved in care and multidisciplinary team meetings are essential factors for interprofessional collaboration. These factors must be taken into consideration to provide valuable, high-quality care to older people residing in long-term care and geriatric.

**Trial registration:**

Not applicable.

**Supplementary Information:**

The online version contains supplementary material available at 10.1186/s12909-024-05291-8.

## Background

Long-term care (LTC) and geriatric rehabilitation (GR) have become increasingly complex in recent years, due to more multimorbidity and complex geriatric syndromes [1, 2]. In LTC, patients often reside permanently in nursing homes, whereas in GR, patients temporarily live on a specifically designed rehabilitation unit within nursing homes, where the care goal is focused on returning home (or moving to a LTC facility if that going home is no longer deemed feasible). Providing valuable, high-quality care for a growing population of older people requires multidisciplinary knowledge, treatment, and appropriate interprofessional collaboration (IPC) [1, 3, 4]. The World Health Organisation defines IPC as: multiple healthcare professionals from different professional backgrounds providing comprehensive services by working with patients, their families, carers, and communities to deliver the highest quality of care across settings [5]. 

Factors influencing IPC can roughly be categorised as interdependent factors related to team performance, sharing information, and organisational conditions [4]. For effective IPC, relationships are essential, not only within the team but also when delivering care to patients and their families [4, 6, 7]. Research by Gittell et al. has shown that effective IPC requires a mutually reinforcing process of communicating and relating for the purpose of task integration [6, 8]. Herewith, all involved individuals, including patients and informal caregivers, can work collaboratively with shared knowledge on shared goals in mutual respect [4, 6, 9]. Their involvement will be enhanced by factors such as communication about preferred roles, having solid relationships, and receiving adequate information from those involved in the care [4, 10–12]. Additionally, effective IPC also requires helpful organisational circumstances, such as supportive relational structures that enhance connections across different groups of people involved in LTC and GR [4, 13]. 

There are still challenges to overcome to accomplish effective IPC with the involvement of all stakeholders (i.e., patients, their informal caregivers, and healthcare professionals) in LTC and GR. So far, only a few studies on IPC have been conducted in GR, and patients and informal caregivers are often not included. Besides, comparative research between LTC and GR is limited. Although both LTC and GR provide complex care for older people, delivered by healthcare professionals from various disciplines, little is known about differences in facilitators of and barriers to IPC between both settings [1, 2]. 

Facilitating IPC requires knowledge of perceived influencing factors in general and during multidisciplinary team meetings (MDTMs). A MDTM is a formal meeting for the purpose of sharing information, discussing the health status of patients, and evaluating the care using standardised patient outcome measures to improve communication [14–17]. Understanding each other’s expertise and conditions to facilitate MDTMs is essential [4, 6, 15]. The MDTM provides an ideal opportunity for all stakeholders to meet, evaluate, and coordinate care. It is also one of the places where IPC happens. When MDTMs are well organised and coordinated, they can enhance the relational and communication processes [6, 7, 18–20]. This improves IPC and the quality of care [6, 15, 21]. 

The primary aim of this study is to examine which facilitators of and barriers to IPC are experienced by patients, informal caregivers, and healthcare professionals involved in LTC and GR. Specifically, a distinction is made between (a) facilitators of and barriers to IPC experienced in general and specifically during MDTMs; and (b) differences between LTC and GR. A secondary aim is to explore if and which patient outcome measures are used in MDTMs.

## Methods

By conducting constructivist research [22, 23], we were able to develop an understanding of the perceived facilitators of and barriers to IPC according to patients, informal caregivers, and healthcare professionals in general and during MDTMs. Both focus groups and individual interviews were used in this qualitative study to make use of the strengths of each method to enhance the understanding of IPC in LTC and GR perceived by all stakeholders [23, 24]. Interviews provide the opportunity to explore subjects in more detail, centred around the unique experience of a participant that can offer comprehensive perceptions [23, 25]. Whereas focus groups reveal how participants respond to group dynamics that may influence their thinking and behaviour. Focus groups also enable participants with similar backgrounds to use their values and norms [23, 25]. The Consolidated Criteria for Reporting Qualitative Research Checklist (COREQ) was used for reporting [26]. 

The Medical Research Ethics Committee Leiden Den Haag Delft (METC LDD) [N22.027] judged the study to be exempt from the Medical Research Involving Human Subjects Act. Participants received a 20-Euro gift card for their participation. Data were collected between March and December 2022.

### Setting

This study was conducted in Dutch nursing homes that provided LTC and GR care to older people with complex diseases and disabilities [27–30]. Patients residing in nursing homes are permanently or temporarily unable to live at home and receive 24/7 care. Patient care is provided by a multidisciplinary team consisting of elderly care physicians, psychologists, speech therapists, occupational therapists, physiotherapists, nurses, healthcare aides, spiritual counsellors, social workers, and activity supervisors. Healthcare teams in Dutch nursing homes are led by elderly care physicians, who work closely together with all other healthcare professionals [28]. GR facilities specifically focus on frail elderly people who have complex multimorbidity and reduced learnability and trainability. Generally, GR patients have a reasonable chance of returning to their homes [2]. A multidisciplinary team specialising in GR delivers treatment and care with a higher intensity than the treatment and care provided in LTC [29]. The length of stay in GR is up to six months, but mostly shorter, after which patients can again participate in society [31]. 

### Participants

First, the scientific research committees of the nursing homes were asked whether the organisation wanted to participate in the study. An information package about the study was sent to chair of the scientific research committee, and an additional appointment with the researcher (AD) was scheduled if needed to explain more about the study. After receiving permission, potential participants were approached by the manager. They received an information letter and informed consent form. After agreement to enrol in the study, purposive sampling [23] was used to ensure the minimal number of participants per group for the focus groups and interviews (see ‘Data analysis’ for more details about the sample size). All participants had to work, reside, or have a relative residing in LTC or GR, and have sufficient knowledge of the Dutch language to participate in a focus group or interview. Additional inclusion criteria for healthcare workers were a minimum age of 18 and working in LTC or GR for longer than three months. Informal caregivers had to be actively involved in their relative’s care.

### Data collection

When the appointment for the interview or focus group was confirmed, participants received the definition of IPC by the World Health Organisation. Also, they received a link to a short online questionnaire about their demographic characteristics. The questionnaire was made and sent via Castor Electronic Data Capture (Amsterdam, Netherlands), an electronic data capture system that helps streamline the process of collecting, storing, and securing data. A paper version was provided for patients and for participants unable to fill in the questionnaire online. If needed, the researcher provided help with filling in the questionnaire.

### Focus groups

Each focus group consisted of at least four participants to enable discussion of the emerging issues [23, 25]. To reduce the possibility of hierarchical influences, we chose to work with separate groups according to the positions in Dutch LTC and GR facilities [28]. Separate focus groups were held for: (1) physicians and therapists; (2) nurses and healthcare aides; (3) managers; (4) patients; and (5) informal caregivers. Participants in a single focus group were all working in the same organisation, however, they could work in different departments or locations.

Focus groups lasted 90 to 120 min and were conducted at the participating organizations. One researcher (AD; male, background in physiotherapy, experience with qualitative research) led the focus groups, assisted by a second researcher (HS; female psychologist with ample experience with qualitative research, or one of the three research interns with a background in medicine or psychology: TV, LS, MD). The second researcher also made field notes.

Researchers used elicitation techniques to facilitate the group discussions and encourage participants to share their ideas [25, 32]. The focus group started with participants being invited to write down facilitators of and barriers to IPC that they experienced. Next, the factors were discussed within the group. The second researcher collected the individual sheets and added those to the transcript of the focus group. Using an inversion technique, participants were then invited to think of hurdles in order to obstruct a MDTM. Following, they were asked how to revolve and solve the hurdles together with the group. The focus group ended with discussing two statements within the group: (1) The use of patient outcome measures during MDTMs enables communication between healthcare professionals, patients, and informal caregivers; and (2) Using patient outcome measures facilitates care.

### Semi-structured interviews

Semi-structured interviews lasted 45 to 60 min and were conducted with participants represented in each of the five groups. Four researchers (AD, TV, LS, and MD) conducted live or online interviews, depending on the participant’s preference. The research interns received elaborate instructions on how to conduct the interviews. The interview topic guide is presented in Supplement 1.

Focus groups and semi-structured interviews were conducted simultaneously. Both the focus groups and semi-structured interviews were audio-recorded and transcribed verbatim. We started coding the focus groups and interviews during data collection to ensure data saturation was reached. The transcript or summary of the focus group or interview was not returned to the participants for a member check. This was partly due to practical reasons (e.g., some participants did not have access to an Internet connection, or due to time constraints). Field notes were made during the focus groups and interviews.

### Data analysis

A sample size of at least 40 participants for ten focus groups (two per group) was deemed sufficient to generate a general idea of the factors perceived by the stakeholders as influencing IPC in LTC and GR [23, 33]. For the semi-structured interviews, a sample of 10–20 interviews corresponding the five groups was deemed sufficient to reach data saturation [23]. 

Data were coded by combining an inductive and deductive approach to a thematic analysis [23, 34, 35]. The coding was performed by a team of four researchers (AD, TV, LS, and MD). Two researchers independently coded the transcripts, followed by a consensus meeting. A third researcher was consulted in case the two researchers could not reach an agreement.

The development of the initial coding tree was based on the themes from a review of IPC in LTC and GR [4]. These themes were: (A) team performance, which involves clarity of roles and goals, attitude and interaction between participants; (B) organisational conditions, which included procedures, resources, and leadership; and (C) information sharing, which consisted of communication between and involvement of participants and the exchange of information. Additional codes were added to the coding tree based on inductive coding. Herewith, we generated data-driven codes in addition to theory-driven codes [34]. 

The six phases of Braun and Clarke for a thematic analysis were followed [34, 36]. During an iterative process, the researchers (AD and HS) searched for relations in codes to develop candidate themes and sub-themes. These themes were discussed between the researchers and the research team to develop an interpretative story. Atlas.ti version 22 facilitated the ordering and structuring of the data [37]. The demographic data of participants was descriptively analysed using SPSS version 25.

## Results

A total of ten focus groups and eighteen semi-structured interviews were conducted in eight facilities providing LTC and GR. A total of 14 patients, 13 informal caregivers, 10 managers, and 22 healthcare professionals participated. The demographics of participants are shown in Table 1.

**Table 1:** Demographics of participants from long-term care and geriatric rehabilitation involved in the study per group and per setting

		Healthcare professionals			Patients	Informal caregivers
		Physicians and therapists	Nurses and healthcare aides	Managers		
Focus groups	LTC	3	6	4	4	6
	GR	3	2	3	5	5
Interviews	LTC	5	1	2	4	1
	GR	1	1	1	1	1
Mean age in years (*SD*)	LTC	35.3 *(6.8)*	51.3 *(12.8)*	50.8 *(8.7)*	80.5 *(10.2)*	72.7 *(9.8)*
	GR	31.5 *(7.9)*	46.3 *(10.1)*	53.3 *(12.9)*	75.3 *(7.2)*	55.5 *(13.8)*
Gender (n male /female)	LTC	0 / 8	0 / 7	0 / 6	2 / 6	4 / 3
	GR	0 / 4	2 / 1	2 / 2	2 / 4	4 / 2
Mean years working in care team (*SD*)	LTC	4.8 *(6.8)*	3.9 *(1.9)*	7.7 *(6.9)*		
	GR	4.8 *(2.9)*	7.0 *(5.6)*	2.7 *(1.5)*		
Mean hours working per week (*SD*)	LTC	22.0 *(7.1)*	29.7 *(6.0)*	33.7 *(7.8)*		
	GR	34.0 *(2.3)*	29.3 *(8.3)*	30.0 *(12.0)*		
Mean length of stay in months (*SD*)	LTC				18.1 *(4.3)*	
	GR				1.3 *(0.6)*	
Mean hours actively involved in patient care per week (*SD*)	LTC					20.1 *(17.1)*
	GR					20.2 *(19.7)*
Mean years actively involved in patient care (*SD*)	LTC					18.5 *(16.5)*
	GR					3.6 *(4.5)*
Relationship to the patient	Partner			LTC		3
				GR		2
	Son/daughter			LTC		2
				GR		2
	Brother/sister			LTC		1
				GR		1
	Son-/daughter-in-law			LTC		1
				GR		0
	Neighbour			LTC		0
				GR		1

*LTC, Long-Term Care; GR, Geriatric Rehabilitation*

### Factors influencing IPC

Patients, informal caregivers, and healthcare professionals reported numerous factors influencing IPC in LTC and GR. These were grouped into two general themes and one specific theme regarding MDTMs. An overview of all themes, categories, and codes is presented in Tables 2 and 3.

**Table 2:** Overview of the themes, categories, and codes of facilitators of and barriers to interprofessional collaboration in general

Potential Themes	Categories	Codes	
		Facilitators	Barriers
*Involvement of patient, informal caregiver, and healthcare professional*	Informing each other	• Clear information exchange between healthcare professionals	• Healthcare professionals do not know who is informal caregiver
		• Balance between sharing information in writing and through conversation	• Informal caregiver is not informed, or not in a timely manner, or is informed retrospectively
		• Up-to-date handover information	• People involved are not or insufficiently informed about the care process
		• Informing family caregivers about policy decisions	• Family caregivers are not or insufficiently informed about the roles of healthcare professionals
		• Clarity about care process among all persons involved (patient, family caregiver and healthcare professional)	• Ignoring family caregiver contribution
	Mutual communication	• Mutual communication among stakeholders	• No or limited communication between persons involved
		• Short lines of communication between professionals in nursing home and hospital care	• No or limited contact between family caregiver and healthcare professional
		• Uniform use of language	• Limited communication between healthcare professionals
		• Listening to each other	
	Participation of all persons involved	• Engaging with each other	• Unclear attitude towards each other
		• Willingness of patient, family caregiver and health care professional to help	• Family caregiver not or not actively involved
		• Open attitude	• Not knowing each other
		• Interest in each other	
		• Positive team atmosphere	
		• Equality of permanent and temporary team members	
	Behaviour and attitude of team members towards each other	• Low-threshold accessibility of healthcare professionals	• Showing no interest in each other•
		• Calling each other to account/giving feedback	• Little or no empathy
		• Showing empathy	
		• Social activities with team to get to know each other	
		• Self-reflection	
	Team members’ expectations towards each other	• Agreement on goals	• Failure to honour agreements
		• Mutually attuned expectations	• No or limited attuning
*Systematic approach to providing care for older people*	Coordinating team procedures	• Clear procedures	• No clear procedures
		• Joint coordination regarding treatment plan	• No or limited coordination between care professionals about their contribution
		• Working in complementary manner to each other	
		• Systematically planned evaluation moments with persons involved	
	Coordinating organisational procedures	• Availability of multidisciplinary health professionals for treatment	• Healthcare professionals having no or limited control regarding treatment process
		• Policy support for collaboration between care professionals	• Unclear policy on procedures

**Table 3:** Overview of the themes, categories, and codes of facilitators of and barriers to interprofessional collaboration specific for multidisciplinary team meetings

Potential Themes MDTM	Categories MDTM	Codes MDTM	
		Facilitators	Barriers
*Organised participation of patient, informal caregiver, and healthcare professional in MDTMs*	Participants in the MDTM	• Patient and/or family caregiver attending the MDTM	• Patient and family caregiver not involved in MDTM
		• Multidisciplinary healthcare professionals attending the MDTM	
		• Decisive MDTM chairperson	
		• Patient-tailored presence of disciplines at MSTM	
	MDTM - Team procedures	• Patient and family caregiver influence on content of MDTM	• Ignoring team agreements regarding MDMT
		• Clarity about MDTM process	• No clear vision about conducting the MDMT
		• Clarity about goal of MDTM	
		• Clarity about chairmanship of MDTM	
	Methodical approach to MDTM	• Planned actions during MDTM	
		• Organized consultation structure for providing feedback from MDTM to patient and family caregiver	

The first theme related to general IPC was ‘Involvement of the patient, informal caregiver, and healthcare professionals’, consisting of the categories: a) participation of patients and informal caregivers; b) behaviour and attitude of team members, c) expectations of team members towards each other, d) the exchange of information, knowledge, and reciprocity in communication.

The second theme was ‘A systematic approach to providing care for older people’. It was composed of the categories: (a) coordinating team procedures, and (b) coordinating organisational procedures.

The theme specific for facilitators of and barriers to IPC during MDTMs was ‘Organised participation of patient, informal caregiver, and healthcare professionals in MDTMs’. This theme was composed of the categories (a) team procedures of MDTMs, (b) working systematically in MDTMs, and (c) participants of MDTMs.

### Themes for IPC in general

#### Involvement of the patient, informal caregiver, and healthcare professional

Participants emphasised that the involvement of all stakeholders, including patients and informal caregivers, is essential for IPC. More specifically, they indicated that the participation, behaviour, and attitude of people in LTC and GR were important influencing factors. Emerging facilitating factors of IPC, such as engaging with each other, having an open attitude towards others, and giving feedback to each other, are discussed by all groups.

*“We have no problem saying things to each other, for example a psychologist who thinks I should approach a patient in a different manner. He will come to me directly: ‘please say it in more in a positive way instead of giving commands’. And that often helps.” Healthcare professional (therapist) - GR in interview 26701*.

According to the participants, engaging with each other also creates a sense of responsibility and responsiveness towards each other. They underlined that connecting with each other enables communication between them.

*“We intentionally pay attention to each other, and our cooperation is improving more and more, so we are increasingly open towards each other. It becomes easier to bring up and discuss collaboration problems.” Healthcare professional (Manager) - GR in focus group 16701*.

However, informal caregivers mainly felt not being involved was a barrier to IPC– for instance, not being informed about potential changes in the care, or unclear involvement of others *–* can be a barrier to IPC.

*“I never see a nurse or therapist when visiting my husband. They never Call or communicate otherwise concerning my husband. We are hardly. Informed because my husband is not able to speak clearly due to his health situation.” Informal caregiver– GR in focus group 16,901*.

*“When I have a complaint about the care of my stepmother, to whom do I go? You see healthcare professionals with blue, lilac, and white uniforms. Some have name tags, others don’t. You don’t know who to turn to because you are insufficiently informed.” Informal caregiver– LTC in focus group 15901*.

Knowing the expectations of team members was also mentioned by participants as an influencing factor. Involvement can be enhanced by factors such as mutual coordination with regard to expectations and having shared goals.

*“The power of taking care of people and doing that together, especially to empower the client. Being in charge in life, looking for goals that are important to that particular person, because I can think up all kinds of stuff, but it has to benefit the person in question.” Healthcare professional (therapist) - LTC in interview 25,702*.

Nevertheless, aligning expectations can be challenging. Examples mentioned were when people do not keep agreements or when different healthcare professionals have different expectations of the goals of a patient.

*“Should someone undergoing rehabilitation be able to make their own bed, or does care staff do it for them?” Patient - GR in interview 26,801*.

Within the theme of ‘involvement of patient, informal caregiver, and healthcare professionals’, factors related to ‘the exchange of information, knowledge, and reciprocity in communication’ were regarded as important for IPC. Participants noted that factors such as sufficient and efficient communication with each other about the care process and listening to each other were enabling factors that can result in a sense of well-being.

*“No matter how busy they are, they take the time to listen to you, I think that’s very important and that feels good.” Patient - LTC in interview 25,810*.

However, when the communication between people involved in LTC and GR is insufficient, it will create discontent among those involved in the care and restrict IPC.

*“This morning my father was informed in like five seconds, your wife is going to move. I think, as her daughter I would like to be there. Why am I not told beforehand?” Informal caregiver - GR in focus group 16,901*.

#### A systematic approach to providing care for older people

Healthcare professionals noticed the coordination of team procedures as important for IPC. They stated, for instance, that when a patient is overburdened, the care for this patient requires adequate coordination between all stakeholders. With this, they can align their involvement with the patient’s needs. They also noticed that IPC is enhanced when team members work in ways that complement each other and use comprehensible methods.

*“I am currently facilitating team collaboration. I don’t do that myself, others do this together and I am the one who makes sure that everyone actually can and will do it. So I walk the wards, am visible, approachable, and I solve things that are mine to solve. But others solve things where they need to.” Healthcare professional (manager) - GR in interview 26702*.

*“Now, we often call the therapist in the morning to say the patient will be 15 minutes late because he is getting dressed. The effort of getting washed and dressed is also often an exhausting activity the patient has to learn, besides the activities learned by a specific therapist, before returning home.” Healthcare professional (nurse)– GR in interview 26,703*.

Participants furthermore emphasised that coordination of organisational procedures is an important overall category within the theme of systematic approach. The availability of supportive organisational policies for IPC was a frequently mentioned an influencing factor. They also indicated the importance of multidisciplinary healthcare professionals having the opportunity to work together in providing care. However, participants also noted the possibility of diverse interpretations in the work process as a potential pitfall. For anyone involved in care, implicit working processes and unclear policies regarding working methods are reported to hinder collaboration and reaching joint objectives.

*“Recently, communication between planning staff and rehabilitation staff has improved by working according to guidelines. We now know better what to expect of each other, which supports the sharing of information even before the patients arrive at the department. This helps to organise to have the right people at the right place at the right moment.” Healthcare professional (nurse)– GR in interview 26703*.

*“And those rules, because the more rules there are, the more leeway you have to bypass them. You need a shared a vision rather than a whole bunch of separate rules.” Patient - GR in interview 26801*.

### Specific theme for MDTMs

#### Organised participation of patient, informal caregiver, and healthcare professionals in MDTMs

Participants specifically emphasised factors influencing IPC during MDTMs. They stated that working with team procedures and using a systematic approach in MDTMs are important. They noticed that a clear process and well-defined goals of MDTMs facilitated IPC during these meetings.

*“In MDTMs, you are digging a little deeper and searching; you specifically look into a patient. Actually brainstorming on how you can best solve potential problems with each other.” Healthcare professional (therapist)– LTC, in interview 25705.*

The participants also revealed that the involvement of a variety of people in MDTMs must be clear. However, they also felt that an unclear vision of team functioning generates uncertainty regarding the involvement of the participants in MDTMs.

*“All disciplines have their contribution during a MDTM. Their input will be discussed by the team. Finally, the team concludes, sets goals, and determines the date on which the patient returns home.” Healthcare professional (therapist)– GR, in interview 26701*.

*“Also a difference in vision. Whether you see the MDTM as no more than chatting for an hour, or that it is where actions and evaluations are agreed upon. That really is a huge difference.” Healthcare professional (healthcare aide) - LTC in focus group 15702*.

In general, there are similarities in many of the factors reported to influence IPC in LTC and GR. However, with regard to MDTMs, differences are reported for organisational procedures, such as a higher frequency of MDTMs in GR (once a week) than in LTC (once every six months). Furthermore, the involvement of patients and informal caregivers during MDTMs is regularly seen in LTC, but not in GR. Here, patients and informal caregivers are mainly informed prior to MDTMs and afterwards.

### Patient outcome measures

An overview of the mentioned patient outcome measures used during MDTMs is shown in Table 4. Standardised patient outcome measures were not often used in MDTMs, according to the participants. Measures mentioned related to the status of physical and psychological functioning and patient safety.

**Table4:** Overview of the mentioned patient outcome measures used in multidisciplinary team meetings in long-term care and geriatric rehabilitation

Patient outcome measures
Activities of Daily Living
Assessment of Motor and Process Skills
Medication management
Barthel Index
Blood pressure
Blood test (blood sampling)
Body Mass Index
Canadian Occupational Performance Measure
Day curves
Decubitus risk score/ Time model
Delirium observational screening
Fall risk
Fluid (intake/output) list
Geriatric Depression Scale
Glucose measurement
Hand squeeze test
Heart rate
Incident report Client
Incident report Staff
Mini-Mental State Examination
Montreal Cognitive Assessment
Neuropsychiatric Inventory
Neuropsychological examination
Pain score
Risk inventories
Short Nutritional Assessment Questionnaire
Temperature
Utrecht scale for the evaluation of clinical rehabilitation
Weight, Bioelectrical Impedance Analysis
6-minute walking test

## Discussion

This study examined perceived facilitators of and barriers to IPC in general and specifically for MDTMs experienced by patients, informal caregivers, and healthcare professionals in LTC and GR. Facilitators of and barriers to IPC in general were classified into two general themes: (1) ‘Involvement of patient, informal caregiver, and healthcare professional’, (2) ‘Systematic approach to providing care for older people’. One specific theme was identified for IPC in MDTMs: ‘Organised participation of patient, informal caregiver, and healthcare professional in MDTMs’. The standardised patient outcome measures were scarcely used during MDTMs.

Effective IPC in LTC and GR is associated with the involvement of patients, informal caregivers, and healthcare professionals. It is person-centred in that it addresses the individual needs of the stakeholders [38–40]. Working together with patients and informal caregivers contributes to person-centred care, which is often mentioned as the golden standard for LTC and GR care [41, 42]. It also enables ‘shared decision-making’ through an open attitude and behaviour and the sharing of information between healthcare professionals, patients, and informal caregivers [42, 43]. However, working with patients and informal caregivers can be challenging [44, 45]. 

To reach a level of collaboration in which all stakeholders work in complementary ways, an open attitude towards others is imperative [4, 9, 43]. An open attitude supports the opportunity to align expectations [46–48]. This enables substantial reciprocity, which is related to a sense of well-being and thus facilitates IPC [47, 49]. However, when, for example, people are not honouring agreements, it may lead to misaligned expectations, which is confirmed in the literature [47]. This may impede involvement and thus hinder IPC.

Besides, we also must consider that being involved with each other can take place at different levels, concern different circumstances, and may change over time, as shown by Lakin (2022) [39]. So, effective IPC warrants continuous attention and regular evaluation by all stakeholders, including the patient and informal caregiver. For a more in-depth insight into facilitators of and barriers to IPC, future studies should examine differences between stakeholders.

The identified theme of a systematic approach (or lack thereof) to providing care is in line with previous studies by Gittell et al. as well as ‘organisational conditions’ in a systematic review on IPC in LTC and GR [8, 46]. Effective coordination will enable healthcare professionals, patients, and informal caregivers to better adapt to often changing situations in the complex care of older people. It can foster their collaboration, which is in line with findings in other studies [50]. This will be supported by having organisational policies about working methods and explicit work processes, as stated in earlier research [51]. So, working systematically should be carefully considered by organisations and teams working in complex care. It enables teams to anticipate frequently changing situations, and anticipatory behaviour benefits group coordination and collaboration [50]. However, more research is needed to examine the role of anticipatory behaviour in LTC and GR with regard to enhancing IPC.

Previous research has shown that a well-organised approach to MDTMs stimulates IPC [9, 52, 53]. Transparency and a systematic approach are beneficial for supporting the involvement of all stakeholders to facilitate person-centred care [2, 9, 46, 54]. It requires the well-coordinated involvement of stakeholders, clear procedures, and aligned communication [6]. Especially in GR, due to the pace of the care process and a noticeable shorter admission time, the involvement of patients and informal caregivers is challenging. Besides, managing expectations in a relatively short period of time appears challenging. To be able to swiftly respond to changing circumstances calls for anticipatory behaviour [50]. However, as a result of an unclear vision of team functioning in MDTMs, collaboration within a group can easily deteriorate [9, 52, 53]. So, to enhance IPC in general, facilities for LTC and GR can benefit from a well-defined context of MDTMs.

Interestingly, we found that standardised patient outcome measures were used scarcely in MDTMs. Although the literature shows it has various beneficial factors such as sharing information, discussing the health status of patients, and evaluating the care, which supports a comparable view of patients’ functional status [55]. In addition, it ensures uniformity in language, which is a facilitator to IPC [4]. These factors can be supportive and align the mutual expectations of patients, informal caregivers, and healthcare professionals. Besides, using patient outcome measures can be supportive of team coordination for the appropriate involvement of healthcare professionals to fit patients’ needs. In this way, LTC and GR can benefit from using patient outcome measures to facilitate IPC in MDTMs and in general. Future studies should examine perceived barriers to using patient outcome measures in MDTMs.

### Strength and limitations

The strengths of this qualitative research include working with a clear definition of IPC, the active involvement of patients, informal caregivers, and healthcare professionals from both LTC and GR, and the use of separate focus groups to provide an opportunity for the various stakeholders to elaborate on their experiences in a setting created to avoid hierarchical influences. Although conducting homogeneous focus groups may have led to missing discussions with mixed participants groups, the semi-structured interviews allowed for an in-depth examination to cover all important topics regarding IPC. It should be noted that this study focused primarily on facilitators of and barriers to IPC. Patient outcome measures to enhance IPC and differences between LTC and GR were discussed less extensively. This study was performed in the Netherlands. In other countries or cultures, where the care for elderly persons is organised differently, other themes may come up. However, the perceived facilitators of and barriers to IPC may still provide useful for optimising IPC in other countries and settings.

## Conclusions

Interprofessional collaboration is necessary for the provision of person-centred, high-quality care in LTC and GR. The complexity of caring for older people residing in these facilities requires coordinated multidisciplinary knowledge, care, and treatment. This necessitates the well-organised collaboration between healthcare professionals, patients, and informal caregivers, both in general and in MDTMs. Enhancing such a collaboration involves a systematic approach with clear policies regarding working methods and effective coordination [6]. Also essential to enable responding to the often changing circumstances is anticipatory behaviour from all stakeholders. Acknowledging the often changing circumstances also means acknowledging that effective coordination is essential [6, 8]. It supports regulating and evaluating the involvement of all stakeholders to meet patients’ needs, which will enhance IPC [40, 46, 47, 54]. Besides to improve care, the use of patient outcome measures in MDTMs may enhance IPC by facilitating the use of a common language, sharing information, which in turn improve team coordination and communication. Thus, interprofessional collaboration in complex care in LTC and GR requires a well-coordinated team consisting of all stakeholders involved to facilitate person-centred high-quality care.

### Electronic supplementary material

Below is the link to the electronic supplementary material.

Supplementary Material 1

## Data Availability

The dataset is available from the corresponding author upon reasonable request.

## List of abbreviations

LTC: Long-Term Care
GR: Geriatric Rehabilitation
IPC: Interprofessional Collaboration
MDTM: Multidisciplinary team meeting
METC LDD: Medical Research Ethics Committee Leiden Den Haag Delft
