# Supplementary material for: Factors influencing interprofessional collaboration in general and during multidisciplinary team meetings in long-term care and geriatric rehabilitation: a qualitative study
Source: BMC Med Educ. 2024 Mar 15;24:285. doi: 10.1186/s12909-024-05291-8 (PMC10941503; doi:10.1186/s12909-024-05291-8)
Supplement: Supplementary file 1 — Supplementary Material 1 [file 12909_2024_5291_MOESM1_ESM.docx]

**Supplement 1.** *The interview topic guide*

FACTORS INFLUENCING INTERPROFESSIONAL COLLABORATION IN

GENERAL AND DURING MULTIDISCIPLINARY TEAM MEETINGS IN LONG-

TERM CARE AND GERIATRIC REHABILITATION: A QUALITATIVE STUDY

Authors; blinded for reviewers

**Interview guide for study on factors influencing interprofessional collaboration**

*Questions for healthcare professionals*

- How would you describe the daily collaboration within your team?
- Can you name any factors that facilitate Interprofessional collaboration (IPC) that you experience in your daily practice?
- Can you name any factors that impede IPC that you experience in your daily practice?
- Can you describe what the multidisciplinary team meeting looks like?
- How do you experience cooperation in the multidisciplinary team meeting (MDTM)?
- Can you name any factors that facilitate IPC that you experience during the MDTM?
- Can you name any factors that impede collaboration that you experience during the MDTM?

*Questions for patients*

1. How do you experience collaboration in the ward?
2. Can you name conditions you experience in the ward that encourage collaboration?
3. Can you name conditions you experience in the ward that impede collaboration?
4. Have you ever attended a MDTM? If so, can you name any circumstances that encourage and/or impede collaboration?

*Questions for family caregiver;*

1. How would you describe your experience of the collaboration in your relatives ward?
2. Can you name conditions you experience in the ward that encourage collaboration?
3. Can you name conditions you experience in the ward that impede collaboration?
4. Have you ever attended a MDTM? If so, can you name any circumstances that encourage and/or impede collaboration?
